# Copper oxide nanoparticles inhibit pancreatic tumor growth primarily by targeting tumor initiating cells

**DOI:** 10.1038/s41598-019-48959-8

**Published:** 2019-08-30

**Authors:** Madeleine Benguigui, Iris S. Weitz, Michael Timaner, Tal Kan, Dvir Shechter, Or Perlman, Sarit Sivan, Ziv Raviv, Haim Azhari, Yuval Shaked

**Affiliations:** 1 Cell Biology and Cancer Science, Rappaport Faculty of Medicine, Technion-Israel Institute of Technology, Haifa, 31096 Israel; 2 Department of Biotechnology Engineering, ORT Braude College, Karmiel, 2161002 Israel; 3 Department of Biomedical Engineering, Technion-Israel Institute of Technology, Technion City, Haifa 3200003 Israel

**Keywords:** Cancer therapy, Cell death

## Abstract

Cancer stem cells, also termed tumor initiating cells (TICs), are a rare population of cells within the tumor mass which initiate tumor growth and metastasis. In pancreatic cancer, TICs significantly contribute to tumor re-growth after therapy, due to their intrinsic resistance. Here we demonstrate that copper oxide nanoparticles (CuO-NPs) are cytotoxic against TIC-enriched PANC1 human pancreatic cancer cell cultures. Specifically, treatment with CuO-NPs decreases cell viability and increases apoptosis in TIC-enriched PANC1 cultures to a greater extent than in standard PANC1 cultures. These effects are associated with increased reactive oxygen species (ROS) levels, and reduced mitochondrial membrane potential. Furthermore, we demonstrate that CuO-NPs inhibit tumor growth in a pancreatic tumor model in mice. Tumors from mice treated with CuO-NPs contain a significantly higher number of apoptotic TICs in comparison to tumors from untreated mice, confirming that CuO-NPs target TICs *in vivo*. Overall, our findings highlight the potential of using CuO-NPs as a new therapeutic modality for pancreatic cancer.

## Introduction

Pancreatic cancer is the fifth deadliest malignancy worldwide. Owing to the asymptomatic nature of the disease and therefore late diagnosis, pancreatic cancer patients have poor prognosis and short survival. In addition, pancreatic cancers are generally unresponsive to standard therapies such as gemcitabine^1^. Mechanisms underlying therapy resistance include a high rate of somatic mutations, transcriptional and post-transcriptional reprogramming, as well as poor distribution of the anti-cancer drug in the desmoplastic pancreatic tumor microenvironment^2^. Currently, a combination of surgery and adjuvant chemotherapy is often used as part of the treatment protocol especially in cases of early diagnosis^2^. However, treatment options are limited, with low success rates. Therefore, new treatment modalities for pancreatic cancer are needed.

Cancer stem cells, or tumor-initiating cells (TICs), represent a small subpopulation of cancer cells within the tumor mass that share common properties with stem cells^3^. Like stem cells, TICs have been shown to support tumor initiation and growth, therefore help tumor progression^3^. Furthermore, TICs are resistant to the majority of treatments, and as such, while anti-cancer drugs kill the bulk of cancer cells within the tumor mass, TICs continue to divide and differentiate, serving as a reservoir of therapy-resistant tumor cells and repopulate the tumor mass^3^. Mechanisms by which TICs resist chemotherapy include quiescent ability^4^, active DNA repair mediated by P21^5^, and increased reactive oxygen species (ROS) scavenging^6^. The latter mechanism is related to the fact that TICs usually contain lower levels of ROS compared to the corresponding differentiated cancer cells^6^. These collective characteristics protect TICs from DNA damage-induced cell death, the mechanism by which chemotherapy targets rapidly dividing cancer cells^7^.

Metal oxide nanoparticles (NPs) represent an emerging treatment for cancer. Their potent and selective cytotoxicity against cancer cells has been demonstrated in several preclinical studies^8,9^. Of the different metal oxide NPs (e.g., ZnO, TiO_2_, Fe_2_O_3_), copper oxide nanoparticles (CuO-NPs) were significantly more toxic in human cell lines than other metal oxide nanoparticles^10^. The antitumor activity of CuO-NPs has been demonstrated preclinically in various cancer types including hepatocarcinoma, lung carcinoma, nasopharynx cancer, breast cancer, cervical carcinoma, and pancreatic cancer^9^. The mechanism underlying the anti-tumor activity of CuO-NPs involves the increase of ROS production and oxidative stress which causes DNA damage, increased death receptor expression and ultimately apoptosis-mediated cell death^11,12^. Nevertheless, CuO-NPs are most prominent as they also possess antibacterial and antifungal activity^13,14^, and can serve as a contrast agent for multimodal (both MRI and UltraSound) imaging^15^. Hence, it can potentially be used as a theranostic agent for the treatment of cancer. However, to date, the anti-tumor effects of metal oxide NPs, and in particular CuO-NP on TICs have not been studied.

Here we show that CuO-NPs directly kill TICs via increased ROS production and mitochondrial damage induction, ultimately leading to cell cycle arrest and apoptosis. Accordingly, treating pancreatic tumor-bearing mice with CuO-NPs inhibits tumor growth, in part by specifically killing TICs. This study provides mechanistic insight into the cytotoxic effects of CuO-NPs, highlighting their therapeutic potential for pancreatic cancer.

## Results

### PANC1 TICs are sensitive to the cytotoxic effects of CuO-NPs

**Figure 1:**
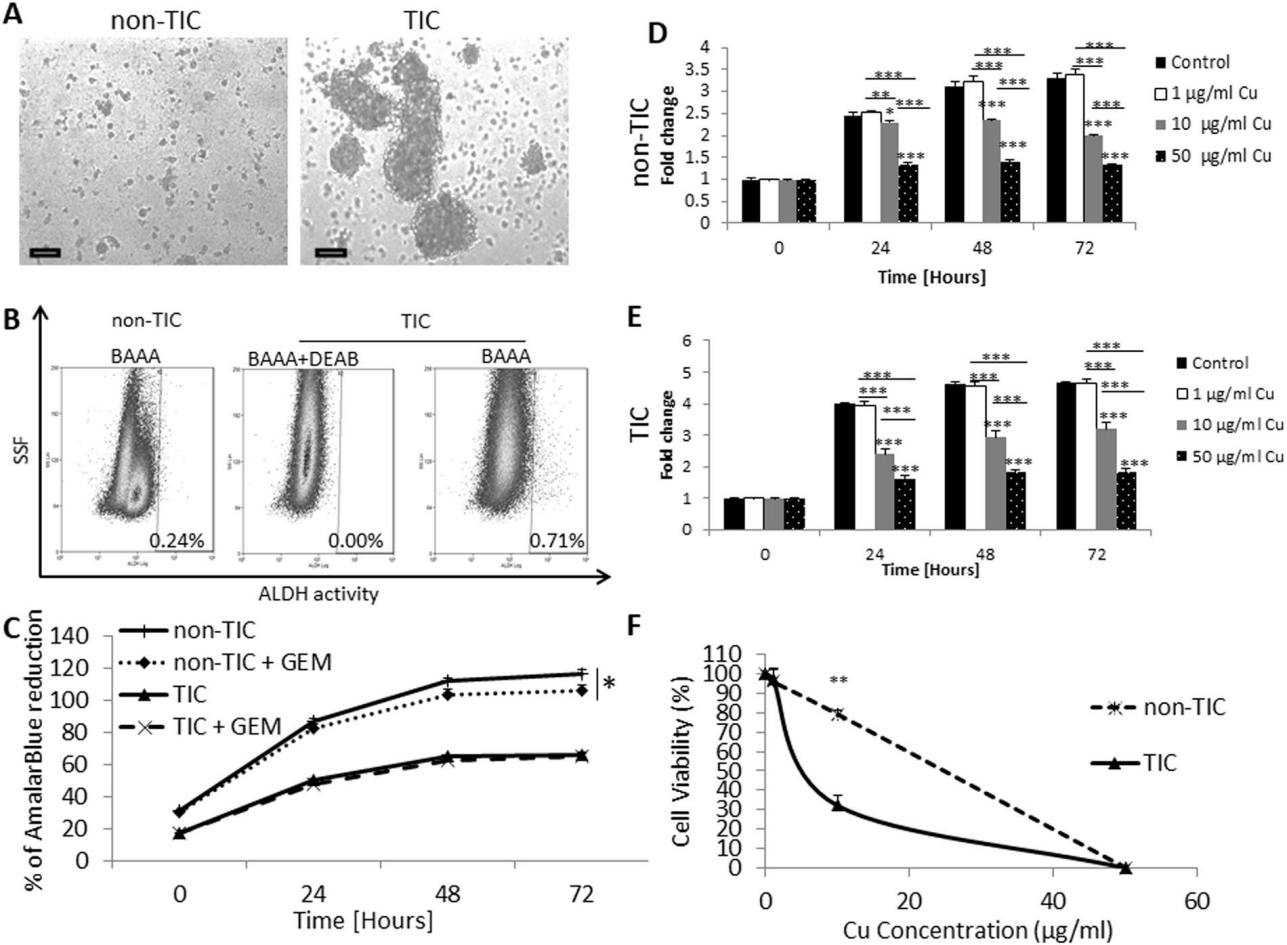
The effect of CuO-NPs on TICs and non-TICs viability. PANC1 human pancreatic carcinoma cells were grown under standard culture conditions (non-TIC) or TIC-enriching conditions (TIC). (**A**) After 14 days, images of cultured cells were captured. Scale bar = 100 µm. (**B**) non-TIC and TIC cultures were analyzed for ALDH activity. Representative dot-plot are shown. (**C**) non-TIC and TIC cultured were exposed to gemcitabine chemotherapy (100 nM for 3 consecutive days), and cell viability was assessed by AlamarBlue assay. (**D**,**E**) Non-TIC (**D**) and TIC (**E**) cultures were treated with escalating doses of CuO-NPs. Cell viability was assessed over time by AlamarBlue assay. Fold change of AlamarBlue reduction is shown. (**F**) IC-50 of CuO-NP was assessed in TIC and non-TIC cultures at the 24 hour time point. Experiments were performed in triplicate, with at least two biological repeats. *p < 0.05; **p < 0.01; ***p < 0.001 as assessed by one way ANOVA followed by Tukey post-hoc test or student t-test when the comparison is between two groups.

It has been previously shown that CuO-NPs are cytotoxic against tumor cells^8^. However, their cytotoxic effect specifically on TICs has not been studied. To characterize the effect of CuO-NPs on TICs, we first generated TIC from PANC1 cell line, and compared them to PANC1 cells cultured in standard conditions, as previously described^16,17^. Hereafter, TIC-enriched and standard PANC-1 cultures are referred to as TICs and non-TICs, respectively. We confirmed that these cultured conditions enriched for TICs and non-TICs, based on sphere forming morphology, increased ALDH activity, and resistance to gemcitabine chemotherapy found in TIC cultured conditions when compared to non-TIC conditions (Fig. 1A–C).

Next, we evaluated the viability of a TIC-enriched culture of the PANC1 cell line in the presence of increasing concentrations of CuO-NPs. For comparison, the effect of CuO-NPs was evaluated in non-TIC conditions. The results in Fig. 1D,E demonstrate that CuO-NPs significantly reduced the cell viability of TICs in a dose-dependent manner, an effect that was also apparent in non-TICs. Notably, a dose response curve at the 24 hour time point shows that the IC50 dosage of CuO-NPs was significantly lower for TICs in comparison to non-TICs (~10 µg/ml vs ~20 µg/ml), demonstrating that TICs are more sensitive to the cytotoxic effects of the agent, at least in the first 24 hours (Fig. 1F). At later time points, TICs and non-TICs exhibited similar sensitivities to CuO-NPs (Fig. 1D,E). Overall, these results demonstrate that at least *in vitro*, TICs are more susceptible than non-TICs to the cytotoxic effects of CuO-NPs.

### CuO-NPs induce apoptosis of TICs

**Figure 2:**
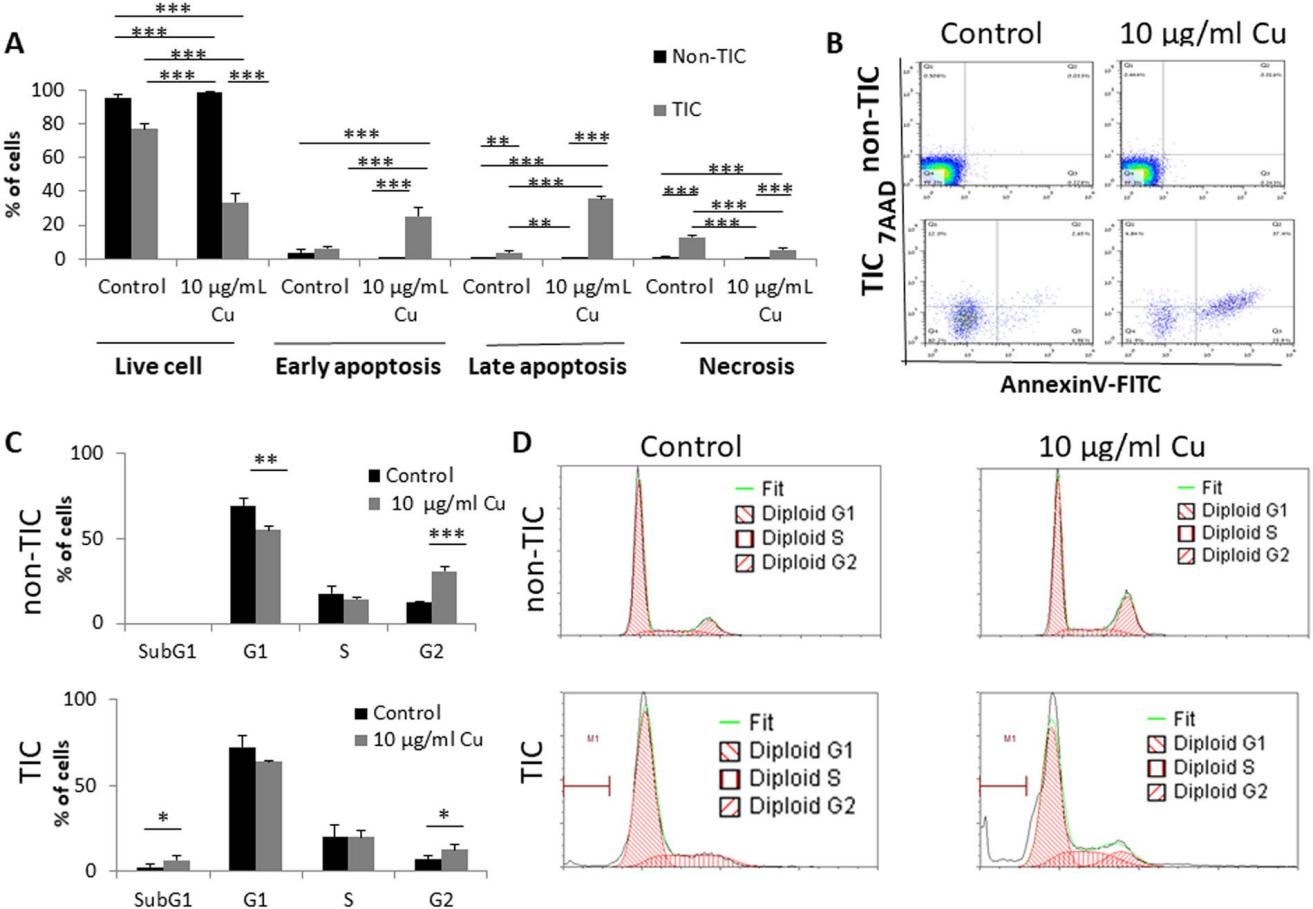
CuO-NPs induce apoptosis of TICs and promote their arrest in Sub-G1 phase. (**A**,**B**) PANC1 cells were grown under standard culture conditions (non-TIC) or TIC-enriching conditions (TIC) in the presence of CuO-NPs (10 µg/ml Cu) for 24 hours. Different stages of cell apoptosis and necrosis were evaluated by 7AAD and Annexin V staining followed by flow cytometry acquisition and analysis. Percentages of cells in each stage are shown in (**A**), and representative flow cytometry plots are shown in (**B**). (**C**,**D**) PANC1 cells were treated as in (**A**,**B**). Cell cycle phases were assessed by flow cytometry. Percentages of cells in each phase are shown in (**C**), and representative flow cytometry histograms are shown in (**D**). Experiments were performed in triplicate, with at least two biological repeats. *p < 0.05; **p < 0.01; ***p < 0.001 as assessed by one way ANOVA followed by Tukey post-hoc test.

We next asked whether the cytotoxic effect of CuO-NPs on TICs is due to induction of apoptosis. To this end, TICs and non-TICs of PANC1 cells were treated with CuO-NPs (10 µg/ml Cu) for 24 hours, conditions under which the difference in viability between the two populations was most apparent (Fig. 1C). The cells were then stained with Annexin V and 7AAD and analyzed by flow cytometry to assess the different stages of apoptosis and necrosis. Early (Annexin V+) and late (Annexin V+/7AAD+) stages of apoptosis as well as the necrotic (Annexin V−/7AAD+) stage were significantly increased in TICs in response to CuO-NP treatment. In contrast, CuO-NP treatment had no effect on any stages of apoptosis in non-TICs at this early time point (Fig. 2A,B).

It has been previously reported that cells undergoing DNA damage usually accumulate in G2 phase, leading to apoptosis (sub-G1 phase)^18^. We therefore evaluated cell cycle phases of TICs and non-TICs in response to CuO-NP treatment. We found a substantial increase in Sub-G1 and G2 phases in TICs exposed to CuO-NPs when compared to an increase only in G2 phase and a decrease in G1 phase in non-TICs. Importantly, the sub-G1 fraction was substantially higher only in the CuO-NP-exposed TICs (Fig. 2C,D). Collectively, these results indicate that under these particular conditions, CuO-NP treatment induces apoptosis in TICs to a greater extent than in non-TICs.

### CuO-NPs elevate ROS levels and reduce mitochondrial membrane potential in TICs

**Figure 3:**
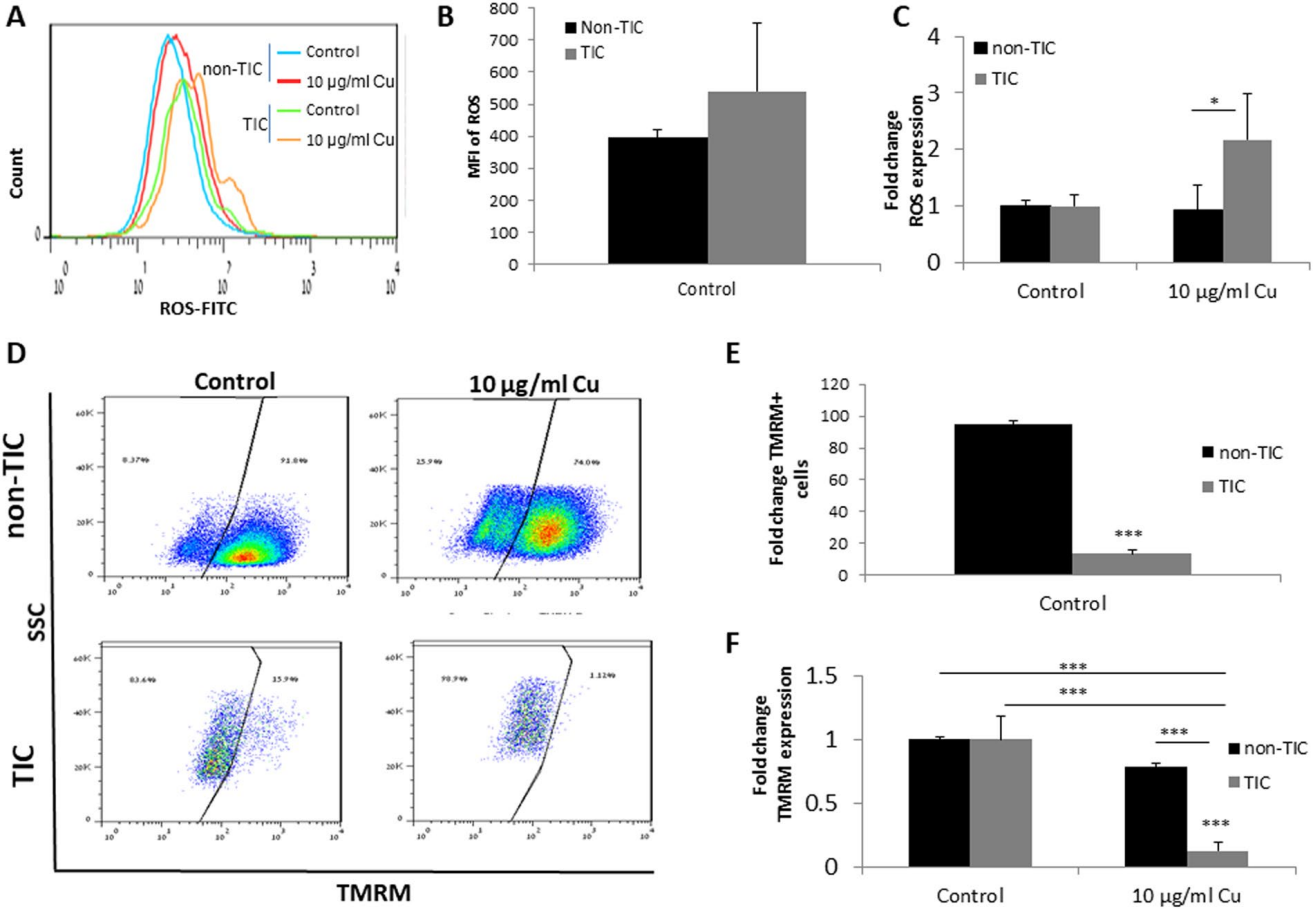
CuO-NPs increase ROS and decrease mitochondrial membrane potential in TICs. (**A**–**C**) PANC1 cells were grown under standard culture conditions (non-TIC) or TIC-enriching conditions (TIC) in the presence of CuO-NPs (10 µg/ml Cu) for 24 hours. ROS levels were evaluated by flow cytometry. A representative flow cytometry histogram is shown in (**A**). Graph of control non-TIC and TIC cultures of mean fluorescent intensity (MFI) of only ROS-positive (FITC+) cells is shown in (**B**), and the fold increase of ROS following exposure to CuO-NPs is shown in (**C**). (**D**–**F**) Cells treated as in (**A**–**C**) were evaluated for mitochondrial membrane potential by TMRM staining followed by flow cytometry acquisition and analysis. Representative flow cytometry dot plots are shown in (**D**). The percentage of TMRM+ cells in control conditions are shown in (**E**), and the fold change in the percentage of TMRM+ cells is shown in (**F**). Experiments were performed in triplicate, with at least two biological repeats. *p < 0.05; ***p < 0.001 as assessed by one way ANOVA followed by Tukey post-hoc test.

ROS are increased in stress conditions, and their accumulation in cells leads to cell apoptosis^19^. We therefore asked whether increased levels of ROS account for CuO-NP-induced apoptosis in TICs of pancreatic carcinoma cells. To this end, we evaluated the levels of ROS in TIC and non-TIC cultures of PANC1 cells, 24 hours after they were exposed to CuO-NPs (10 µg/ml Cu). As shown in Fig. 3A,B, untreated control TICs exhibited higher ROS activity than non-TICs as measured by mean fluorescence intensity, when taking into account only ROS positive cells, although these results did not reach statistically significance. Importantly, following exposure to CuO-NP, a significant increase in the level of ROS was found in TICs only (Fig. 3A,C). These results may suggest that TICs are prone to the effect of CuO-NP leading to ROS-induced apoptosis.

Increased cellular ROS levels leading to apoptosis are often caused due to mitochondrial damage^20^. To test whether mitochondrial dysfunction accounts for increased ROS levels in response to CuO-NPs, we evaluated mitochondrial membrane potential (ΔΨm) in TICs and non-TICs cultured for 24 hours in the presence or absence of CuO-NPs (10 µg/ml Cu). TIC and non-TIC cultures demonstrated a different level of ΔΨm under control conditions, in which TICs demonstrated low ΔΨm, thereby projecting mitochondrial dysfunction (Fig. 3D,E). Yet, when normalizing their levels, we found that CuO-NP treatment induced a significant reduction in ΔΨm in both cell populations. However, the effect was much more pronounced in TICs in comparison to non-TICs (~80% vs. ~20% reduction; Fig. 3D,F). Taken together, these results indicate that CuO-NPs promote cell apoptosis by increasing ROS and decreasing ΔΨm, effects which are more substantial in TICs than in non-TICs.

### CuO-NPs inhibit pancreatic tumor growth in mice

**Figure 4:**
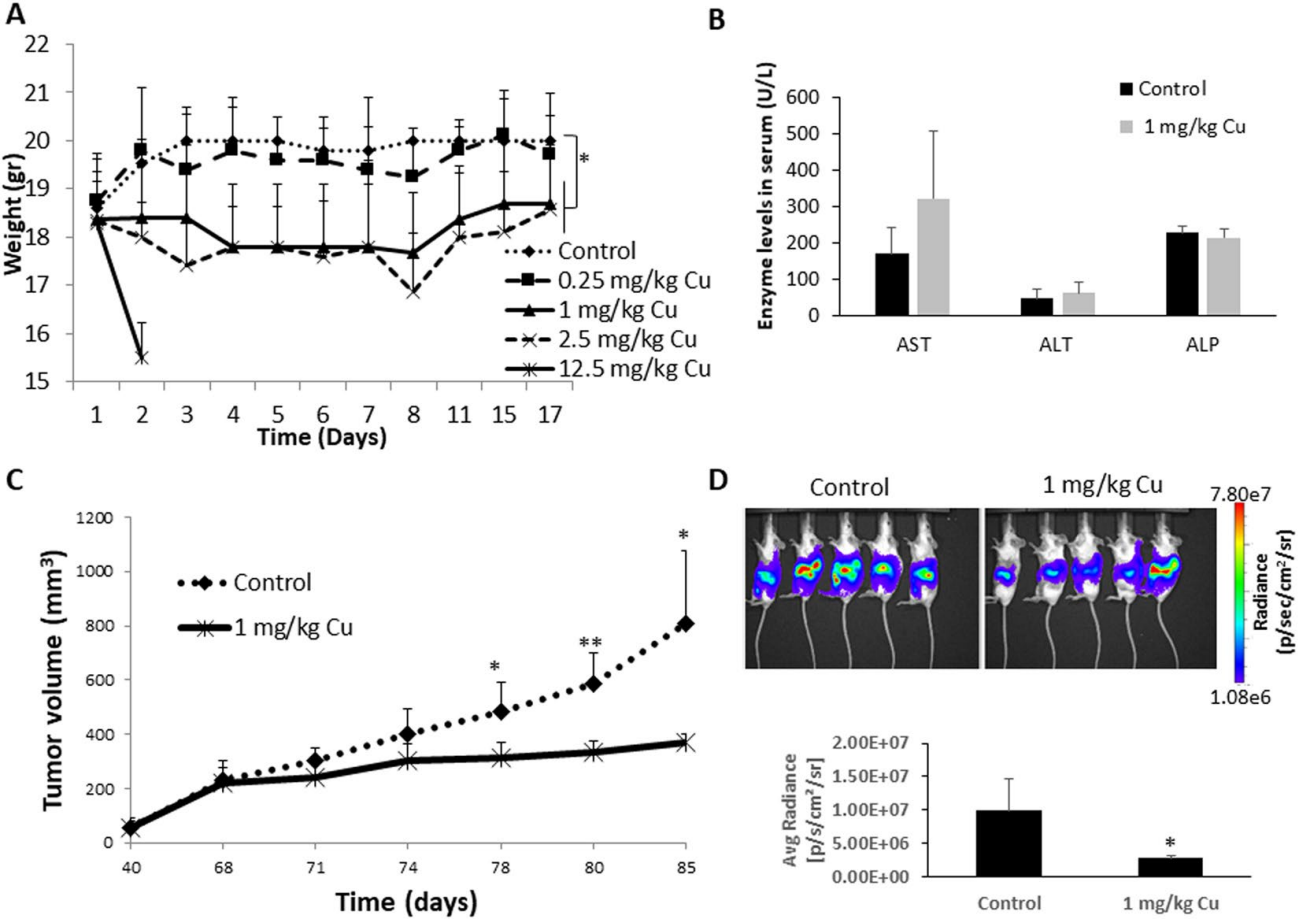
CuO-NPs inhibit pancreatic tumor growth *in vivo*. (**A**) Non-tumor bearing BALB/c mice were treated with CuO-NPs at doses ranging from 0–12.5 mg/kg Cu (n = 5 mice/group). Body weight was assessed over time. (**B**) Non-tumor bearing BALB/c mice were treated with CuO-NP at a dose of 1 mg/kg Cu (n = 4 mice/group). After 7 days, mice were bled by cardiac puncture, and serum was evaluated for biomarkers of hepatotoxicity. (**C**) PANC1 cells were subcutaneously implanted into the flanks of 6 week old NOD-SCID mice (n = 5 mice/group). When tumors reached 200 mm^3^, mice were injected with vehicle control or CuO-NPs (1 mg/kg Cu) daily for 7 sequential days. Tumor growth was monitored over time. (**D**) PANC1 cells tagged with luciferase, were orthotopically implanted in 6 week old NOD-SICD mice (n = 5 mice/group). On week 2, mice were injected with vehicle control or CuO-NPs (1 mg/kg Cu) daily for 7 sequential days. Tumor bioluminescence was assessed by IVIS on week 5, and bioluminescence measurements were plotted. Of note, mouse 5 in the treatment group is an outlier and removed from the quantification. *p < 0.05; **p < 0.01 as assessed by two-tailed student t test.

We next investigated the therapeutic advantage of CuO-NP *in vivo* in a mouse pancreatic tumor model in mice. First, we evaluated the toxicity of CuO-NPs in non-tumor bearing mice treated with vehicle or escalating doses of CuO-NPs (0.25, 1, 2.5 or 12.5 mg/kg Cu) for 7 sequential days. Based on changes in body weight over a period of 17 days, mice treated with the lowest dose of CuO-NPs (0.25 mg/kg Cu) did not exhibit signs of toxicity. Higher doses (1 and 2.5 mg/kg Cu) resulted in weight loss of approximately 10%. Notably, mice receiving the highest dose (12.5 mg/kg Cu) were euthanized within 2 days due to a substantial reduction in body weight (Fig. 4A). After two weeks, hematotoxicity was assessed by quantifying the number of neutrophils, lymphocytes and total white blood cell (WBC) counts. No significant changes were identified in any hematological parameter tested, although a trend towards reduced WBC count was found only in the dose of 2.5 mg/kg Cu (Table 1). Of note, it appears that lymphocyte and neutrophil counts were slightly increased in higher doses of CuO-NPs, but such effects were limited to small number of mice, thus this could explain why the results did not reach statistical significance. Elevated levels of bone marrow derived cells in response to cytotoxic drugs have already been reported^21,22^, which could explain these effects when using CuO-NP therapy. Furthermore, we also evaluated hepatotoxicity using serum biomarkers, and found that there are no significant differences in AST, ALT and ALP in the serum of mice treated with 1 mg/kg when compared to untreated control mice (Fig. 4B). Overall, these results indicate that CuO-NPs are tolerable at a dose of 0–1 mg/kg Cu.

**Table 1:** Hematotoxicity in mice treated with CuO-NPs.

Drug dose (mg/kg Cu)	Total WBC count (/µL PB)	Lymphocyte count (/field of BS)	Neutrophil count (/field of BS)
0	5450 ± 2207	41 ± 15	12 ± 6
0.25	4875 ± 2390	39 ± 18	19 ± 3.5
1	5070 ± 1175	67 ± 16	22.7 ± 5.5
2.5	3117 ± 506	51 ± 23	37 ± 41.2

PB - peripheral blood, WBC – white blood cells, BS – blood smear.

To test whether CuO-NPs inhibit pancreatic tumor growth *in vivo*, PANC1 cells were subcutaneously implanted into non-obese diabetic severe combined immunodeficient (NOD-SCID) mice. When tumors reached 200 mm^3^, mice were treated with vehicle or CuO-NPs at a dose of 1 mg/kg Cu for 7 sequential days. Tumor growth was assessed for additional 10 days post-treatment. The results in Fig. 4C demonstrate that CuO-NP treatment significantly delayed tumor growth in comparison to control. These results were confirmed when we used a more clinically relevant tumor model when mice were orthotopically implanted with PANC1 cells tagged with luciferase into the pancreas as described in Methods (Fig. 4D).

**Figure 5:**
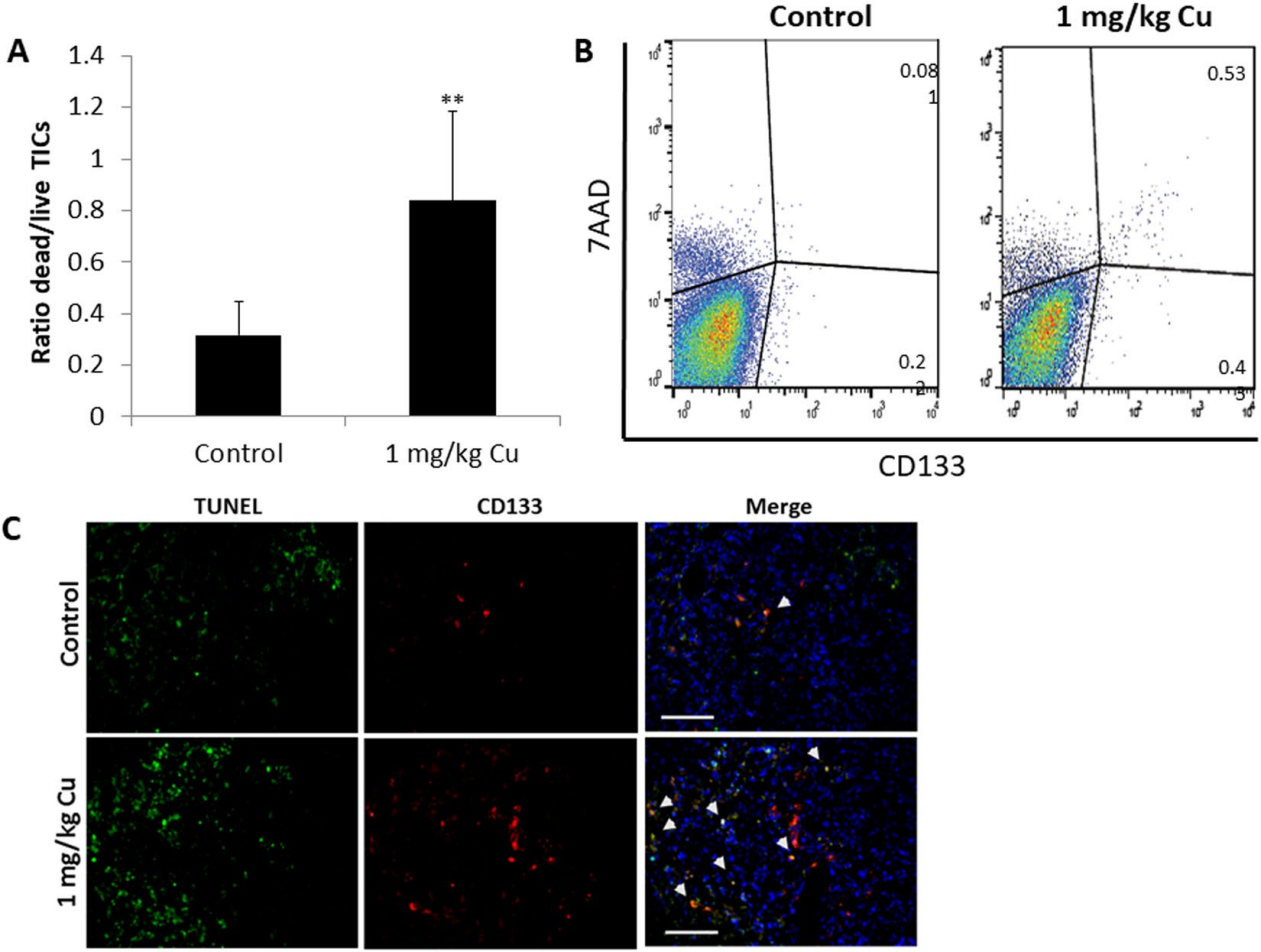
CuO-NPs target TICs *in vivo*. PANC1 cells were subcutaneously implanted into the flanks of 7 week old NOD-SCID mice (n = 5 mice/group). When tumors reached 200 mm^3^, mice were injected with vehicle control or CuO-NPs (1 mg/kg Cu) daily for 7 sequential days. At endpoint, mice were euthanized, and tumors removed. (**A**,**B**) Tumors were prepared as single cell suspensions. Apoptotic/dead TICs (7AAD+; CD133+) were quantified by flow cytometry. The ratio of dead:live TICs is shown in (**A**), and representative flow cytometry plots are shown in (**B**). (**C**) Tumors were cryosectioned and immunostained for CD133 (red) and TUNEL (green) to detect TICs and apoptotic cells, respectively. Nuclei were stained with DAPI (blue). Representative images are shown. Scale bar = 50 µm. **p < 0.01 as assessed by two-tailed student t test.

Next, when control mice reached endpoint (Fig. 4C), mice from both groups were euthanized and tumors were excised. Flow cytometry analysis of tumor suspensions revealed an increased ratio of apoptotic over live TICs in tumors from CuO-NP-treated mice in comparison to control mice (Fig. 5A,B). Similarly, an increased number of apoptotic TICs was observed in immunofluorescently stained tumor sections from CuO-NP-treated mice (Fig. 5C). Overall, our findings demonstrate that CuO-NPs have an anti-tumor activity primarily due to their cytotoxic effects against TICs.

## Discussion

Pancreatic cancer is considered one of the most lethal cancers. Recent studies have demonstrated that pancreatic tumors are highly enriched with a TIC population that is responsible for cancer initiation, progression, metastasis, and chemoresistance^23^. TICs govern several properties that allow them to resist chemotherapy. It has been shown that TIC characteristics such as enhanced DNA-repair capacity, quiescent properties, superior detoxifying enzymes, as well as overexpression of anti-apoptotic proteins and enhanced drug-efflux pumps contribute to drug resistance^24,25^. Furthermore, Hermann and colleagues have shown that CD133+ pancreatic cancer cells are enriched in tumors after exposure to gemcitabine chemotherapy and are likely to resist therapy in comparison to CD133- negative cells^26^.

Previous studies by us and others have demonstrated that fibroblasts and mesenchymal stem cells (MSCs), in response to chemotherapy, are recruited to the treated tumor site and secrete factors which enrich for TICs^16,27^. Inhibiting such factors in combination with chemotherapy resulted in inhibition of TIC enrichments thereby increasing the likelihood of sensitive tumors to the chemotherapy due to the lack of TICs^16,27^. Here we present an alternative treatment modality that targets TICs in desmoplastic tumors, such as pancreatic cancer. We demonstrate that CuO-NPs specifically target the TIC population, and thus have a potential to be used as an anti-cancer drug. It would be therefore interesting to study the effect of CuO-NPs on the cross talk between tumor cells and cells of the tumor microenvironment such as MSCs and fibroblasts and their impact on TICs.

Here we suggest a possible biological process which can explain the susceptibility of TICs to CuO-NP cytotoxic activity. We demonstrate that already in control conditions TIC cultures contain higher intracellular ROS levels than non-TIC cultures, suggesting that they are more vulnerable to oxidative stress than non-TIC cultures. Treatment with CuO-NPs causes a substantial increase in ROS in TICs, leading to a cell killing effect. These results are counterintuitive in light of previous published studies. Specifically, it has been shown that TICs generally maintain low levels of ROS, due in part, to increased activity of antioxidant enzymes or ROS scavenging molecules^28^. In addition, ROS levels were found to be higher in ovarian and liver non-TIC cultures in comparison to TIC cultures^29,30^. We assume that CuO-NPs specifically inhibit the ability of TICs to maintain low ROS levels. This, in turn, causes a reduction in ΔΨm, leading to apoptosis^9^. Indeed, we found that even in control conditions, TICs display higher levels of apoptotic cells than non-TICs, probably due to the fact that the cultured cells are a mixed population of TICs and non-TICs, as described in Methods. These results are in line with the fact that ΔΨm levels in our system model, were lower in TICs when compared to non-TICs, and such effects were increased in response to CuO-NP exposure for the first 24 hours. Yet, at later time points, cell viability of non-TIC cultures is reduced to the same extent as TIC cultures in the presence of CuO-NP. Therefore, CuO-NPs may be cytotoxic to both TIC and non-TIC populations depending on the duration of therapy exposure.

In accordance with our *in vitro* findings, we demonstrate that CuO-NP treatment delays tumor growth in two mouse models of pancreatic cancer implanted ectopically or orthotopically. These therapeutic effects were demonstrated by the killing effects of CuO-NPs on both TIC and non-TIC populations as demonstrated by flow cytometry and imaging analyses, suggesting that CuO-NP is cytotoxic to both TICs and non-TICs, regardless of the percentage of TICs found in the tumor. Importantly, CuO-NP at the dose of 1 mg/kg Cu did not result in general toxicity represented by body weight, hematotoxicity and hepatotoxicity. We found a slight weight loss (~10%), and no significant change in neutrophils, lymphocytes and total WBCs at the 1 mg/kg Cu dose. These effects are in contrast to chemotherapy drugs which usually cause myelosuppression^31^. It should be noted that NPs may sometimes cause inflammation, therefore, it can increase neutrophil count in peripheral blood as previously demonstrated^32^; however, such effects were not observed in our experimental setting. Yet, in order to increase the dose of CuO-NP above the 1 mg/kg threshold, it would be of interest to evaluate whether other CuO-NP formulations, which specifically target tumor cells and not host cells, exhibit reduced toxicity in high doses. In this regard, Perlman *et al*., demonstrated that CuO-PLGA-NPs, have advantageous ultrasound imaging properties in non-tumor bearing mice in comparison to CuO-NPs^15^. As PLGA can mask the cytotoxic activity of CuO-NP, it could serve as a future candidate that combines lower toxicity, imaging and therapeutic ultrasound properties. It would be of interest to evaluate the distribution of CuO-NP in different organs in order to study its toxic effects, though in our experimental setting no significant changes were found in serum biomarkers for hepatotoxicity. However, the question remains whether other derivatives of CuO-NP can differentially distribute to specific organs and therefore reduce the cytotoxicity of the drug.

In summary, we demonstrate that CuO-NPs have an anti-tumor effect, in part by targeting the TIC population in tumors. These anti-tumor activities are related to the cytotoxicity of CuO-NPs as they induce ROS activity and mitochondrial dysfunction. Our findings highlight the potential of CuO-NP as a treatment modality for TIC-enriched tumors such as pancreatic tumors.

## Methods

### Ethic statement

All animal experiments and methods used were performed in accordance with the guidelines and regulations of the Technion, and approved by the Animal Care and Use Committee of the Technion - Israel Institute of Technology (Haifa, Israel, Ethic number IL-089-07-2016).

### Cell culture

PANC1 human pancreatic ductal adenocarcinoma cell line (obtained from the American Type Culture Collection) was cultured in Dulbecco’s modified eagle’s medium (DMEM, Sigma-Aldrich, Israel) supplemented with 10% fetal bovine serum (FBS, Biological Industries, Israel), 1% L-glutamine, 1% sodium pyruvate, and 1% streptomycin-penicillin-neomycin solution (Biological Industries). The cells were used within 6 months of resuscitation, and were periodically tested to be mycoplasma-free. Cells were cultured at 37 °C in a humidified atmosphere containing 5% CO_2_.

### TIC enriched cultures

TIC-enriched cultures of PANC1 cells were generated by culturing PANC1 cells under non-adherent tumor sphere conditions, containing specific growth factors as previously described^17^. Briefly, monolayer cells grown under standard culture conditions (as above) were harvested with 0.25% trypsin-EDTA and washed twice with PBS. Subsequently, cells were resuspended in sphere media. Sphere medium consisted of serum-free DMEM/F-12 medium supplemented with 10 µg/ml insulin, 100 µg/ml transferrin, 40 ng/ml sodium selenite solution, 0.4% BSA, 20 ng/ml EGF and 10 ng/ml bFGF as previously described^17^. After 10–14 days in culture, cells formed spherical colonies. Supplemental growth factors were added to the sphere cultures every other day. To confirm TIC and non-TIC populations, at day 14, images of spheres were captured using bright field microscope (LEICA DMI6000B, Germany), and the cells were then analyzed by flow cytometry for ALDH activity using the ALDEFLOUR kit (StemCell Technologies) following the manufacturer’s instruction and as previously described^16^. DEAB was used for negative gating. In all experiments, cells were maintained in 100 mm culture dishes (Nunc) in a humidified incubator at 37 °C in 5% CO_2_.

### Preparation of CuO-NPs

The CuO-NPs were used “as-synthesized” and without further surface modifications. The acetate ligands generated during the synthesis, provided satisfactory passivation against aggregation yielding high colloidal stability. CuO-NPs were synthesized as previously described^13^. Briefly, copper acetate (0.545 g, Sigma Aldrich, Israel) was dissolved in 150 ml of deionized water. Acetic acid (0.525 g, Daejung Chemicals, Korea) was then added and the mixture was brought to boiling under vigorous stirring. Subsequently, sodium hydroxide (0.6 g, Bio-Lab Ltd., Israel) was carefully added. After 1 min of reflux, the mixture was cooled to room temperature. The CuO-NPs were then isolated by centrifugation (4000 rpm, 10 min) and washed twice with water. CuO-NPs were redispersed in deionized water or sterile saline for further use. The Cu content was quantified by inductively coupled plasma with optical emission spectroscopy (ICP-OES) analysis, using the 720-ES ICP optical emission spectrometer (Varian). Size and shape of the CuO-NPs were examined by cryogenic transmission electron microscopy (cryo-TEM, FEI Talos 200 C TEM operated at 200 keV).Hydrodynamic size distribution and surface charge of the particles were determined by dynamic light scattering (DLS) and Zeta potential measurements, respectively (ZetaSizer Nano ZS, Malvern). The characteristics of CuO-NPs are summarized in Table 2.

**Table 2:** The Characteristics of CuO-NPs in water. TEM- Transmission Electron Microscope, DLS- Dynamic Light Scattering.

Core size by TEM	~7 nm
Hydrodynamic size(Z-average) measured by DLS	124 nm
Polydispersity Index measured by DLS	0.224
Zeta potential	+37.8 ± 5.14 mV

### Cell viability assay

Cell viability was assessed using the metabolic indicator dye AlamarBlue (AbD Serotech Ltd., Oxford, UK) as previously described^33^. Briefly, standard or TIC enriched sub-confluent PANC1 cultures were re-plated (1200 cells/well in a 96-well plate) in their designated medium containing CuO-NPs (0–10 µg/ml Cu) supplemented with 10% AlamarBlue reagent. In some experiment gemcitabine (100 nM) was added to TIC and non-TIC PANC1 cells, and cell viability was assessed for 3 consecutive days. Absorbance was measured in a plate reader (TECAN M200, Männedorf, Switzerland) using 570 nm and 600 nm wavelengths as a reference. The percentage of AlamarBlue reduction during the 24–48 hour time frame was calculated and corrected to the absorbance values of negative controls. In some control experiments, all cells were grown in the same medium (TIC medium) in order to achieve similar culture conditions for the analysis of cell viability. All experiments were carried out in triplicate.

### Cell cycle and apoptosis assays

Standard or TIC-enriched PANC1 cultures were treated with CuO-NPs (0–10 µg/ml Cu) for 24 hours. For the analysis of apoptosis, cells were detached and subsequently immunostained with anti-prominin-1 (CD133, 1:250, Macs MiltenyiBiotec, Germany) and Annexin V-FITC antibody in the proper binding buffer (MBL, Japan), as recommended by the manufacturer. Next, 7-Aminoactinomycin D (7-AAD, Sigma-Aldrich, USA) was added to the samples at a final concentration of 1 µg/ml. For the analysis of cell cycle, cells were fixed with 70% ethanol, and subsequently stained with 40 μg/ml propidium iodide (PI, BioLegend) in the presence of 10 μg/ml RNase (Sigma Aldrich, Israel). Cells were acquired by CyanADP flow cytometer (Beckman Coulter, Nyon, Switzerland) and data was analyzed by FlowJo software7.6. (San Diego, CA).

### ROS activity assay

ROS activity was determined using a ROS detection kit (Enzo Life Sciences, Enzo Life Sciences, NY, USA) in accordance with the manufacturer’s instructions. Briefly, cells from standard or TIC-enriched PANC1 cultures were seeded in a six-well plate (1 × 10^6^ cells per well). Cells were treated with CuO-NPs (0–10 µg/ml Cu) for 24 h. Subsequently, cells were washed and resuspended in ROS detection solution. As a positive control, cells were treated with apocynin. Subsequently, cells were immunostained with anti-CD133 antibodies to detect TICs, and with 7AAD to discriminate between live and dead cells as previously described^34^. In some control experiments, both culture types were grown under TIC conditions and CD133 was used to distinguish between TIC and non-TIC populations. Cells were acquired by flow cytometry and analyzed by FlowJo software as above.

### Mitochondrial membrane potential measurement

To detect mitochondrial damage, the fluorescent probe tetramethyl rhodamine methyl ester (TMRM, Sigma-Aldrich, Israel) was used to evaluate loss of mitochondrial membrane potential. For this, standard and TIC-enriched PANC1 cultures were exposed to CuO-NPs (0–10 µg/ml Cu) for 24 h. Subsequently, TMRM was added at a final concentration of 250 nM for 40 min at 37 °C. The cells were centrifuged and washed in PBS. Cells were then immunostained with anti-CD133 antibodies to detect TICs and with 7AAD to discriminate between dead and live cells. Cells were acquired by flow cytometry and analyzed by FlowJo as above.

### Toxicity analysis in mice

Naïve 8–10 week old BALB/c mice were intravenously injected with increasing doses of CuO-NPs (0–250 mg/kg Cu, n = 5 mice/cohort) daily for 7 sequential days. Body weight was assessed every day for 2 weeks. To measure hematotoxicity, after two weeks (at endpoint), mice were bled from the retro-orbital sinus, and blood smears were prepared. Smears were stained with Wright-Giensa (Sigma-Aldrich, Israel). Briefly, dried blood slides were immersed for 30 sec in a Coplin jar containing approximately 50 ml Wright-Giensa stain solution. Subsequently, the slides were placed for 5 min in a new Coplin jar containing PBS. Finally, slides were dried thoroughly before examination by light microscopy (Leica Microsystems, Inc. Wetzlar, Germany) to determine the number of lymphocytes and neutrophils per designated field. White blood cells were counted by hemocytometer. To measure blood biomarkers for hepatotoxicity, serum from the treated mice as above were analyzed for aspartate aminotransferase (AST), alanine aminotransferase (ALT), and alkaline phosphatase (ALP) biomarkers in the hematology laboratory at Rambam Health Care Campus (Haifa, Israel).

### Pancreatic tumor models and treatment protocol

PANC1 (5 × 10^6^) cells were subcutaneously injected into the flanks of 6-week-old female NOD-SCID mice (Harlan, Israel). Tumor volume was assessed regularly with Vernier calipers using the formula width^2^ × length × 0.5. When tumors reached 200 mm^3^, the mice were intravenously injected via the retro-orbital sinus with 1 mg/kg Cu or vehicle control for 7 sequential days. At endpoint, when tumors in control mice reached a size of 1500 mm^3^, mice were euthanized, and tumors were resected. Tumors were processed for histopathology and flow cytometry as described below. To obtain an orthotopic pancreatic tumor model, PANC1 cells (5 × 10^5^) tagged with luciferase, were injected to the pancreas of 6-week-old NOD-SCID mice, after the abdominal cavity of the mice was exposed to locate the pancreas and subsequently sutured. Tumor size was monitored by IVIS 200 (PerkinElmer), as previously described^16^. Treatment was initiated on week two for 7 sequential days. Mice were sacrificed at endpoint.

### Flow cytometry analysis of tumors

Resected tumors from mice were prepared as single-cell suspensions as previously described^33^. Single cells were immunostained with anti-human HLA (BioLegend, USA) and anti-human CD133 (Macs Militenyi Biotec) antibodies in accordance with the manufacturer’s instructions. In some experiments, 7AAD was used to discriminate between live and dead cells. At least 200,000 events were acquired using a Cyan ADP flow cytometer and analyzed with FlowJo software as described above.

### Immunohistochemistry

To identify TICs within pancreatic tumors by histology, tumor cryosections (10μm) were immunostained with PE-conjugated antibodies against human prominin-1 (CD133, 1:200, Macs MiltenyiBiotec). Apoptotic cells were assessed using a TdT-mediated dUTP-X nick end labeling assay kit (TUNEL, Roche, Switzerland) in accordance with the manufacturer’s instructions. Tumor sections were analyzed with a fluorescent microscope (LEICA DFC7000T, Germany).

### Statistical analysis

Data are presented as mean ± standard deviation (SD). Statistically significant differences were assessed by one-way ANOVA, followed by Tukey post-hoc test (when comparing between more than two groups) using GraphPad Prism 6 software (La Jolla, CA, USA). When applicable, estimate of variance was performed and statistical significance comparing only two sets of data was determined by two-tailed Student’s t-test. All *in vitro* experiments were performed at least twice in three biological replicates. In the *in vivo* experiments, 5 mice per group were used unless indicated otherwise. Significance was set at values of p < 0.05, and designated as follows: *p < 0.05; **p < 0.01; ***p < 0.001.
